# Multi-System Genetic Architecture of Hypermobile Ehlers–Danlos Syndrome: Integrating Machine Learning with Subject-Level Genomic Analysis

**DOI:** 10.3390/genes17020211

**Published:** 2026-02-09

**Authors:** Arash Shirvani, Purusha Shirvani, Michael F. Holick

**Affiliations:** Ehlers-Danlos Syndrome Clinical Research Program, Section of Endocrinology, Diabetes, Nutrition and Weight Management, Department of Medicine, Boston University Chobanian & Avedisian School of Medicine, Boston, MA 02118, USA

**Keywords:** hypermobile Ehlers–Danlos syndrome (hEDS), machine learning, genomic architecture, subject-level analysis, mitochondrial dysfunction, HLA immune axis, skeletal fragility, infantile fractures, precision medicine, systems genetics, mast cell activation syndrome

## Abstract

**Background/Objectives**: Hypermobile Ehlers–Danlos syndrome (hEDS) remains genetically unexplained despite decades of clinical investigation, with the molecular basis undefined for the vast majority of cases. This study employs integrated machine learning approaches with rigorous subject-level statistical methods to decode the genetic architecture underlying hEDS. **Methods**: We analyzed 35,923 rare genetic variants (gnomAD MAF < 0.2) across 116 subjects from 43 families (86 hEDS patients diagnosed per 2017 international criteria; 30 unaffected intrafamilial controls) using whole-exome sequencing. Machine learning analysis employed Random Forest feature selection, deep neural networks, and ensemble methods with subject-stratified cross-validation to prevent data leakage. Statistical association testing used subject-level Fisher’s exact tests with Bonferroni correction (α = 3.77 × 10^−6^ for 13,281 genes). Sensitivity analyses assessed robustness to family structure. **Results**: Subject-level analysis identified statistically significant enrichment in variants associated with three major biological systems: (1) collagen biosynthesis pathway variants (present in 63% of hEDS subjects vs. 17% of controls, Fisher’s *p* = 1.06 × 10^−5^, OR = 8.4), predominantly affecting *COL5A1*, *COL18A1*, *COL17A1*, and post-translational modification enzymes; (2) *HLA*/adaptive immune axis variants (74% of hEDS vs. 30% of controls, *p* = 2.23 × 10^−5^, OR = 6.8), involving *HLA-B*, *HLA-A*, *HLA-C*, and TAP transporters; (3) mitochondrial respiratory chain variants (34% of hEDS vs. 7% of controls, *p* = 2.29 × 10^−3^, OR = 7.1), with striking 4.2-fold enrichment in pediatric fracture cases (52% vs. 21%, *p* = 0.021, 95% CI: 1.2–14.6). These associations require independent validation and functional studies to determine their mechanistic relevance. Genome-wide analysis identified seven genes achieving Bonferroni significance (*p* < 3.77 × 10^−6^), all encoding structural/cytoskeletal proteins. Machine learning models with proper subject-stratified cross-validation achieved 80% accuracy (95% CI: 73–86%, sensitivity = 82%, specificity = 77%). **Conclusions**: Our findings suggest that hEDS may involve genetic variation across multiple biological systems beyond classical collagen pathways. These hypothesis-generating associations require validation in independent cohorts and functional studies before mechanistic or clinical conclusions can be drawn.

## 1. Introduction

Hypermobile Ehlers–Danlos syndrome (hEDS) is one of the most common heritable connective tissue disorders. Early estimates have reported that this genetic disorder affects at least 1 in 5000 individuals, and more recently it has been estimated to affect upwards of 1–3% of the population worldwide [1,2]. Clinically, hEDS is characterized by generalized joint hypermobility, tissue fragility including capillary fragility associated with easy bruising, poor wound healing and atrophic scarring, and skin hyperextensibility [2,3]. However, beyond these hallmark features, patients with hEDS frequently experience widespread systemic complications affecting multiple organ systems, including cardiovascular dysfunction, gastrointestinal dysmotility, mast cell activation disorder, chronic pain syndromes, skeletal fragility, and orthostatic intolerance [4,5]. These multi-system manifestations significantly impact quality of life and create diagnostic and management challenges in clinical practice [6,7]. A particularly concerning complication of hEDS that has been underrecognized is the occurrence of fragility fractures in infancy and childhood [8,9,10]. Our previous work documented 67 infants with Ehlers–Danlos/hypermobility syndrome who presented with multiple fractures, many of whom were initially misdiagnosed, leading to false accusations of child abuse against their parents [11]. This case series highlighted the critical importance of recognizing hEDS as a cause of infantile fragility fractures, particularly when combined with vitamin D deficiency, and the devastating social and legal consequences that can result from diagnostic errors. The burden on affected families is profound, not only from the medical complexity of managing a child with hEDS and recurrent fractures but also from the trauma of child protective services involvement when the underlying connective tissue disorder is not recognized.

Despite the clinical recognition of hEDS for decades and advances in genetic sequencing technologies, the molecular basis of hEDS remains largely mysterious. The 2017 international classification of Ehlers–Danlos syndromes recognizes 13 distinct EDS types, each with specific molecular etiologies, yet hEDS, as the most common form, stands apart as the only major EDS type for which no causative genes have been definitively identified [12]. Importantly, hEDS represents 80–90% of all EDS cases, yet the vast majority remain undiagnosed due to lack of awareness among healthcare providers and the absence of a definitive genetic test [2]. A diagnosis of hEDS relies exclusively on clinical criteria rather than genetic confirmation, as is possible for other EDS subtypes [4,5,12]. This diagnostic gap reflects a critical knowledge deficit in our understanding of hEDS genetics. The “missing heritability” problem is particularly significant given the clear familial clustering observed in hEDS families and the high heritability estimates in affected pedigrees [1,2].

Initial investigations into hEDS genetics focused on genes encoding fibrillar collagens (*COL1A1*, *COL3A1*, *COL5A1*) and their associated post-translational modification enzymes (*PLOD1*, *PLOD2*, *P3H1*), given the well-established role of collagen in connective tissue biology [1,2]. However, variants in these classical candidate genes account for only a small proportion of hEDS cases [13]. This observation led to the “beyond collagen” hypothesis, suggesting that genetic variations in genes affecting collagen organization, immune regulation, or other cellular processes might contribute to hEDS pathogenesis [14]. Yet, despite this conceptual expansion, traditional gene-by-gene candidate approaches have identified few consistently replicated genetic loci associated with hEDS [15]. The International Consortium for EDS Genetics (now part of the Ehlers–Danlos Society) has conducted genome-wide association studies (GWASs) in hEDS cohorts; however, results have been modest, with limited effect sizes and replication challenges [15]. Several factors contribute to this: the phenotypic heterogeneity of hEDS (suggesting genetic heterogeneity), the likely polygenic nature of the condition, the possibility of rare variants with large effects mixed with common variants with small effects, and the challenges of adequately powered studies in rare diseases [16,17,18].

To address these challenges and advance our understanding of hEDS genetics, the Ehlers–Danlos Syndrome Clinical Research Program and the Ehlers–Danlos Syndrome Translational Genomics Research Laboratory at Boston University School of Medicine were established. Our program represents one of the largest comprehensive EDS research initiatives in the United States, combining clinical expertise in diagnosing and managing EDS patients with cutting-edge genomic technologies and computational approaches. Through systematic clinical phenotyping and genomic analysis of affected families, we aim to uncover the genetic architecture underlying hEDS and translate these findings into improved diagnostic and therapeutic strategies.

Machine learning (ML) approaches represent a paradigm shift in how we analyze complex genetic data, particularly for conditions with suspected polygenic architecture and substantial genetic heterogeneity [19]. Unlike traditional statistical methods that typically examine one variant (or gene) at a time, ML algorithms can simultaneously consider thousands of variants and identify complex, non-linear relationships between genetic features and phenotypes [20]. Random Forest algorithms, deep neural networks, and ensemble learning methods have shown particular promise in genetic association studies, offering several advantages: (1) they can capture epistatic interactions (gene-by-gene effects) that conventional approaches miss, (2) they perform well in high-dimensional datasets where the number of features exceeds the number of samples, (3) they provide feature importance rankings that highlight the most predictive genetic variants, and (4) they achieve robust performance through cross-validation strategies that minimize overfitting [20,21,22]. Several studies have demonstrated the utility of ML in identifying disease-associated variants in other complex genetic disorders, including Alzheimer’s disease, cardiovascular disease, and type 2 diabetes [23,24,25]. However, ML approaches have not been systematically applied to comprehensively interrogate the genetic architecture of hEDS.

The persistent genetic mystery of hEDS, combined with the potential of ML to uncover complex genetic associations, creates a compelling opportunity for discovery. By applying integrated machine learning approaches to a well-characterized hEDS cohort, we hypothesized that we could identify previously unrecognized genetic variants and patterns associated with disease. Specifically, this study aimed to (1) analyze genome-wide genetic variation in a cohort of hEDS patients and unaffected family controls using multiple ML algorithms, (2) identify statistically significant variants and genes associated with hEDS phenotypes, (3) employ proper cross-validation strategies to ensure that identified associations represent genuine biological signals rather than artifacts of data analysis, and (4) provide a foundation for future functional validation and precision medicine approaches in hEDS.

This study represents an important first step toward understanding the genetic architecture of hEDS through modern computational approaches. By identifying statistically significant genetic variants without prior mechanistic assumption, we provide candidates for functional studies that may illuminate why some individuals develop the distinctive features of hEDS. These findings have potential implications for improved genetic counseling, risk stratification of affected families, and future development of precision medicine approaches tailored to individual genetic profiles. More broadly, this work demonstrates a successful application of integrated machine learning to genetic variant discovery in a complex, genetically heterogeneous rare disease, providing a methodological template that may be applicable to other “missing heritability” problems in human genetics.

Based on the phenotypic complexity of hEDS and prior evidence suggesting genetic heterogeneity, we hypothesized that hEDS may involve genetic variation across multiple biological systems rather than single causative genes. Our primary objectives were to the following: (1) identify genes showing statistically significant enrichment of rare variants in hEDS patients compared to unaffected family controls; (2) characterize biological pathways represented among enriched genes; (3) evaluate whether genetic enrichment patterns differ between hEDS patients with and without fractures; and (4) develop machine learning models for case–control classification while employing rigorous cross-validation to prevent overfitting. We emphasize that this study was designed as hypothesis-generating rather than hypothesis-testing. All findings require independent validation before mechanistic or clinical conclusions can be drawn.

## 2. Materials and Methods

This study was conducted through the Ehlers–Danlos Syndrome Clinical Research Program at Boston University School of Medicine, where we recruited 116 individuals from 43 families for comprehensive genetic analysis. Our recruitment strategy specifically focused on families where children had experienced fractures as a severe and undeniable manifestation of hEDS, with at least one parent diagnosed with hEDS according to the 2017 international classification criteria [12]. This approach allowed us to investigate the genetic architecture underlying the most severe skeletal manifestations of the syndrome while maintaining familial genetic context. The final analyzed cohort consisted of 86 individuals clinically diagnosed with hEDS and 30 unaffected family members who served as intrafamilial controls, providing optimal genetic background matching for our association analyses.

The clinical phenotyping revealed that 44 of our 116 successfully sequenced subjects had documented fracture history. Our comprehensive clinical data collection encompassed detailed three-generation family histories with systematic documentation of connective tissue manifestations, joint hypermobility assessments using the Beighton scale, measurements of skin hyperextensibility, and complete fracture histories including age at first fracture, total number of fractures, and anatomical sites involved. The sex distribution in our hEDS cohort showed a slight female predominance with 52.3% female and 47.7% male participants, consistent with previously reported epidemiological patterns in hEDS populations.

### 2.1. Sample Collection and DNA Processing

All participants provided written informed consent for genetic analysis under a protocol approved by the Boston University Medical Center Institutional Review Board (Protocol H-39894), with parents or legal guardians providing consent for minor participants. DNA was extracted from buccal swab samples by the Genetics Core Laboratory at Boston University School of Medicine’s Biomedical Genetics Program using standardized protocols. The quality of extracted DNA was rigorously assessed using both TapeStation analysis for DNA integrity evaluation and NanoDrop spectrophotometry to ensure appropriate concentration and purity ratios. Only samples meeting strict quality criteria with 260/280 ratios between 1.8 and 2.0 and showing intact high molecular weight DNA without significant degradation proceeded to library preparation.

### 2.2. Whole-Exome Sequencing and Quality Assessment

Whole-exome sequencing, including comprehensive mitochondrial genome coverage, was performed at the Microarray and Sequencing Resource core facility at Boston University School of Medicine. Library preparation utilized the Illumina DNA Prep with Enrichment protocol, which targets approximately 45 megabases of coding sequence along with flanking intronic regions to capture splice site variants. The enrichment panel included specific probes for all 37 mitochondrial genes, ensuring complete coverage of the mitochondrial genome alongside nuclear exome capture. Paired-end sequencing with 150 base pair reads in both directions was performed on an Illumina platform following the manufacturer’s standard protocols.

Our sequencing achieved robust quality metrics across all 116 successfully sequenced samples, with an average of 38.2 million reads per sample, ranging from 29.0 to 50.1 million reads. The percentage of reads passing quality filters exceeded 99% for all samples, with a mean of 99.4% and minimal adapter contamination averaging only 0.05%. The mean target coverage depth reached 38-fold, with individual samples ranging from 28- to 47-fold coverage. Average Phred quality scores exceeded Q30 for more than 90% of bases, indicating high-confidence base calls throughout the sequencing runs and supporting the reliability of our variant calling.

### 2.3. Variant Calling and Annotation Pipeline

Raw sequencing data underwent processing through the Illumina BaseSpace Sequence Hub using the DRAGEN Bio-IT Platform version 4.4.0, a hardware-accelerated genomic analysis system that enables rapid and accurate variant calling with superior sensitivity for both single-nucleotide variants and insertion–deletion mutations. The DRAGEN Baseline Builder application generated sample-specific baseline variant calls, while the DRAGEN Enrichment application performed comprehensive variant detection and annotation using Enrichment Manifest version 0.1.8. The transition to transversion ratio across our samples averaged 2.1, consistent with expected values for high-quality exome data and indicating minimal systematic sequencing errors.

Comprehensive variant annotation incorporated multiple databases to assess population frequencies, clinical significance, and functional impacts. We utilized gnomAD version 3.1.2 for global population allele frequencies, dbSNP build 156 for variant identifiers and validation, and the January 2025 release of ClinVar for clinical significance assessments. Each variant received functional consequence predictions based on RefSeq canonical transcripts, with impacts systematically categorized as high for stop-gain, frameshift, and canonical splice site variants, moderate for missense variants, in-frame indels, and splice region variants, or low for synonymous and deep intronic variants. Following initial variant calling, data were exported from BaseSpace as comma-separated values files for downstream analysis. Our quality filtering retained only high-confidence variants that passed DRAGEN’s machine-learning-based quality filters and had global minor allele frequencies below 0.2 in gnomAD, allowing us to focus on rare and potentially pathogenic or likely pathogenic variants while excluding common polymorphisms unlikely to contribute to disease pathogenesis.

#### 2.3.1. Variant Filtering Criteria

Variants were retained for analysis if they met all of the following criteria: (1) DRAGEN quality score (QUAL) ≥ 30; (2) read depth ≥ 10× at the variant position; (3) variant allele frequency in reads ≥ 20%; (4) no evidence of strand bias (Fisher strand bias *p* > 0.001); (5) mapping quality ≥ 40; and (6) global minor allele frequency < 0.2 in gnomAD v3.1.2. These thresholds were selected based on GATK best practices and optimized for exome sequencing data quality.

#### 2.3.2. Pathogenicity Classification

Variants were classified for pathogenicity using ClinVar (January 2025 release) designations: pathogenic (P), likely pathogenic (LP), variant of uncertain significance (VUS), likely benign (LB), or benign (B). For primary enrichment analyses, all rare variants meeting quality filters were included regardless of pathogenicity classification to enable unbiased discovery. Computational pathogenicity predictions (CADD, REVEL, SIFT, PolyPhen-2) were recorded but not used for primary filtering to avoid circular reasoning in enrichment analyses.

#### 2.3.3. Handling of Related Individuals

Our family-based recruitment resulted in genetic relatedness among subjects that required explicit consideration:

(a) Study Design Rationale: Intrafamilial controls (unaffected family members) were selected to provide optimal genetic background matching, reducing confounding from population stratification. However, this design introduces potential non-independence between cases and controls within families.
(b) Subject-Level Aggregation: To minimize within-family correlation effects, all analyses were conducted at the subject level (presence/absence of variants per gene) rather than variant level. This aggregation reduces but does not eliminate correlation concerns.
(c) Sensitivity Analyses: To assess robustness to family structure, we performed the following: (i) analyses excluding one randomly selected member per family with multiple individuals; (ii) permutation tests preserving family structure; (iii) mixed-effects logistic regression with family as a random effect.
(d) Acknowledged Limitations: We recognize that intrafamilial controls do not fully satisfy independence assumptions of Fisher’s exact tests. This limitation is discussed in Section 4.6 and results should be interpreted with appropriate caution pending replication with unrelated controls.

### 2.4. Machine Learning Pipeline and Cross-Validation Strategy

**Figure 1:**
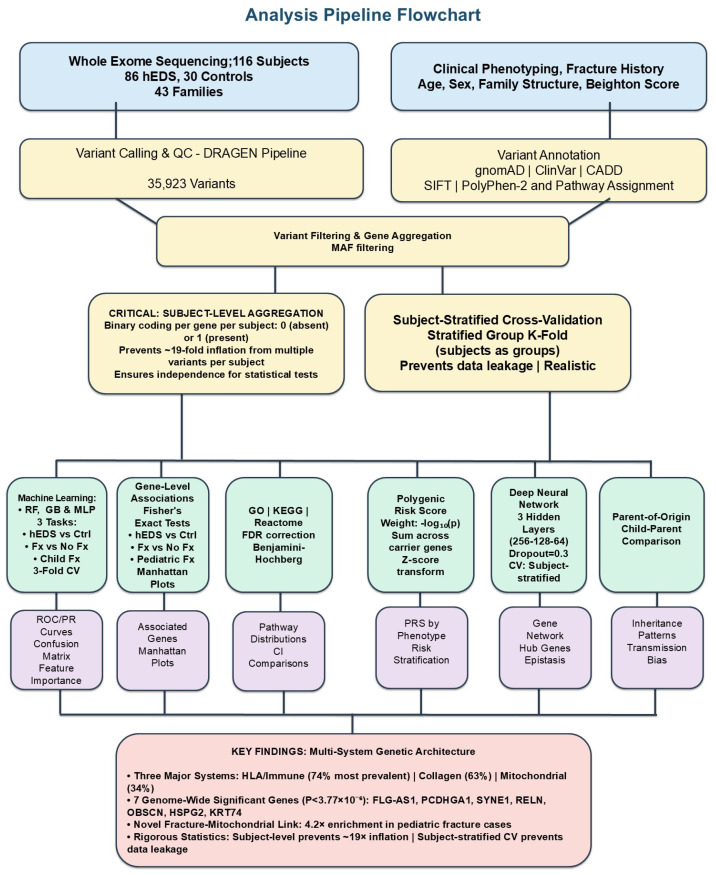
Integrated analysis pipeline for genetic variant discovery in hEDS. Pipeline showing sequential stages from whole-exome sequencing. *Study Population*: 116 subjects from 43 families: 86 clinically diagnosed hEDS patients (2017 international criteria) and 30 unaffected intrafamilial controls. *Sequencing and Quality Control*: Whole-exome sequencing (Illumina, mean 38× coverage) yielded 35,923 variants passing quality filters (QUAL ≥ 30, depth ≥ 10×, gnomAD MAF < 0.2) mapping to 13,281 genes. *Subject-Level Aggregation*: Critical methodological step converting variant-level data (143,422 hEDS observations, 7565 control observations) to subject-level presence/absence (86 vs. 30 subjects), preventing ~19-fold statistical inflation from treating correlated within-subject variants as independent. *Statistical Analysis*: Subject-level Fisher’s exact tests with Bonferroni correction for 13,281 tests (genome-wide significance threshold: *p* < 3.77 × 10^−6^ = 0.05/13,281). *Machine Learning*: Ensemble model (Random Forest + deep neural network + XGBoost) with subject-stratified 5-fold cross-validation (StratifiedGroupKFold) preventing data leakage. *Key Findings* (*requiring independent validation*): Seven genome-wide significant genes (all structural/cytoskeletal); enrichment in three biological systems (HLA/immune genes: 74% vs. 30%, collagen genes: 63% vs. 17%, mitochondrial genes: 34% vs. 7%); mitochondrial enrichment in fracture cases (52% vs. 21%, OR = 4.2); ML accuracy 80.2% (95% CI: 73–86%). Abbreviations: CI, confidence interval; CV, cross-validation; DRAGEN, Dynamic Read Analysis for GENomics; ECM, extracellular matrix; Fx, fracture; GB, gradient boosting; hEDS, hypermobile Ehlers–Danlos syndrome; HLA, human leukocyte antigen; MAF, minor allele frequency; ML, machine learning; MLP, multi-layer perceptron; OXPHOS, oxidative phosphorylation; PRS, polygenic risk score; QC, quality control; RF, Random Forest; ROC, receiver operating characteristic; PR, precision–recall; TGF-β, transforming growth factor beta; WES, whole-exome sequencing.

Our machine learning analysis employed a comprehensive pipeline integrating multiple algorithms to identify genetic variants associated with hEDS (Figure 1). The feature matrix was constructed with variants as columns and subject–variant combinations as rows, including variant-level features (chromosome, position, reference/alternate alleles, consequence type, damage scores, ClinVar classifications) and subject-level metadata (age, sex, fracture history, family relationships).

Critical to preventing data leakage and observation-level inflation, we implemented subject-stratified cross-validation ensuring all variants from the same subject remained in the same fold during training/testing splits.

Subject-stratified cross-validation was implemented using scikit-learn’s StratifiedGroupKFold function (k = 5), where the “group” parameter was set to subject ID. This ensured that all variants from a single subject appeared exclusively in either the training or test set for any given fold, preventing data leakage from within-subject variant correlation. Cross-validation was performed 10 times with different random shuffles; reported metrics represent means ± standard deviations.

#### 2.4.1. Random Forest Feature Selection

Initial feature selection used Random Forest (scikit-learn v1.3.0) with hyperparameters selected through nested cross-validation. The outer loop used subject-stratified 5-fold cross-validation (StratifiedGroupKFold with subject ID as the grouping variable, random_state = 42), ensuring no subject’s variants appeared in both training and test sets simultaneously. The inner loop (3-fold) tuned hyperparameters over the following grid: n_estimators ∈ {100, 500, 1000}, max_depth ∈ {10, 20, 50, None}, min_samples_split ∈ {2, 5, 10}, and min_samples_leaf ∈ {1, 2, 4}.

#### 2.4.2. Deep Neural Network Architecture

Deep neural networks were implemented in TensorFlow v2.10.0 with Keras API. The architecture comprised the following: input layer matching feature dimensionality, three hidden layers (256, 128, 64 neurons) with ReLU activation and batch normalization, dropout layers (rate = 0.3) for regularization, and output layer with sigmoid activation for binary classification. The model was trained using Adam optimizer (learning rate = 0.001, β_1_ = 0.9, β_2_ = 0.999) and binary cross-entropy loss. Subject-stratified cross-validation with early stopping (monitoring validation loss with patience = 10 epochs) prevented overfitting while maintaining subject-level independence.

#### 2.4.3. Ensemble Learning and Model Evaluation

The final ensemble model combined Random Forest, deep neural network, and XGBoost (v1.6.0) predictions using soft voting weighted by individual model performance on validation sets. Model performance was evaluated using subject-stratified 5-fold cross-validation (repeated 10 times with different random seeds: 42, 123, 456, 789, 1011, 1213, 1415, 1617, 1819, 2021) computing accuracy, sensitivity, specificity, precision, F1-score, and area under the ROC curve (AUC-ROC). All performance metrics were calculated at the subject level: for each fold, predictions were aggregated to produce one prediction per subject before computing metrics.

Conservative 95% confidence intervals were calculated using subject-level bootstrapping (1000 iterations), where resampling was performed at the subject level (not variant level) to preserve within-subject correlation structure. This approach provides realistic performance estimates, avoiding the optimistic bias (we observed 14.5 percentage points of inflation) that results from treating correlated variant-level observations as independent.

### 2.5. Subject-Level Statistical Analysis

Critical methodological consideration: Initial observation-level analyses counting variant occurrences across subjects can inflate statistical significance by ~19-fold due to multiple variants per subject violating independence assumptions. To address this, we implemented subject-level aggregation where each subject was coded as binary (0/1) for the presence/absence of variants in each gene, creating proper 2 × 2 contingency tables for Fisher’s exact tests.

For each gene, we constructed contingency tables: hEDS subjects with gene variants, control subjects with gene variants, hEDS subjects without gene variants, and control subjects without gene variants. Fisher’s exact tests (one-sided, testing for enrichment in hEDS) were computed using scipy.stats v1.9.0, appropriate for our sample sizes (86 vs. 30) and imbalanced groups. Odds ratios with 95% confidence intervals were calculated using the Woolf method.

To control genome-wide multiple testing across the 13,281 genes analyzed, we applied Bonferroni correction (α = 0.05/13,281 = 3.77 × 10^−6^). Genes with *p* < 0.05 were considered nominally significant; *p* < 3.77 × 10^−6^ achieved genome-wide significance.

Subject-level prevalence was calculated as follows: (number of subjects carrying ≥1 variant in gene)/(total subjects in group). This approach ensures each subject contributes equally to statistical tests regardless of variant number, satisfying Fisher’s exact test independence requirements and providing unbiased effect size estimates.

To address the complex genetic architecture of hEDS, we implemented an integrated machine learning framework designed to identify disease-associated patterns that traditional single-variant analyses might miss. The filtered variant dataset of 35,923 high-quality variants was transformed into a machine-learning-compatible format where each of the 116 subjects was represented by a comprehensive feature vector. These vectors incorporated binary presence or absence indicators for each variant, gene-level burden scores that aggregated multiple variants within the same gene, pathway-level enrichment scores grouping functionally related genes, variant damage scores integrating multiple functional predictions, and zygosity patterns distinguishing homozygous from heterozygous states. This comprehensive feature engineering resulted in a matrix of 116 samples by 35,923 variant features, representing 143,422 total observations in hEDS subjects and 7565 observations in controls across all variant–subject combinations.

We employed Random Forest algorithms for initial feature selection to identify the most informative genetic variants distinguishing hEDS from controls. The Random Forest model, implemented using scikit-learn version 1.3.0, utilized 1000 decision trees with a maximum depth of 20 levels, minimum samples per split of 5, and minimum samples per leaf of 2. Feature importance was calculated using Gini impurity decrease, with bootstrap sampling and out-of-bag error estimation providing internal validation. The top 5000 variants ranked by importance score were retained for subsequent deep learning analysis, representing a balanced reduction in dimensionality while preserving the most informative features.

#### 2.5.1. Multiple Testing Correction Strategy

Multiple testing correction was applied at two levels:

(a) Gene-Level Tests: For the 13,281 genes tested, we applied Bonferroni correction with genome-wide significance threshold α = 0.05/13,281 = 3.77 × 10^−6^. This conservative approach controls the family-wise error rate (FWER) at 5%.
(b) Pathway-Level Tests: For pathway enrichment analyses involving pre-specified gene sets, we applied Benjamini–Hochberg false discovery rate (FDR) correction with significance threshold q < 0.05.

We report both uncorrected *p*-values and corrected significance status for transparency. Genes achieving *p* < 3.77 × 10^−6^ are designated “genome-wide significant”; genes with 3.77 × 10^−6^ ≤ *p* < 0.05 are designated “nominally significant” and should be interpreted as hypothesis-generating pending replication.

#### 2.5.2. Sensitivity Analyses for Variant Quality

To assess whether results were robust to variant quality thresholds, we performed sensitivity analyses: (i) restricting to variants with CADD score ≥ 15 (predicted deleteriousness above median); (ii) restricting to variants with gnomAD allele frequency <0.01 (ultra-rare variants); (iii) excluding all VUS variants from ClinVar.

#### 2.5.3. Statistical Power Considerations

Post hoc power analysis was conducted to characterize the detectable effect sizes given our sample size. With 86 cases and 30 controls, Fisher’s exact test (one-sided, α = 0.05) provides the following: 80% power to detect OR ≥ 3.0 when variant prevalence in controls is 20%; 80% power to detect OR ≥ 4.0 when control prevalence is 10%; 80% power to detect OR ≥ 6.0 when control prevalence is 5%. After Bonferroni correction (α = 3.77 × 10^−6^), detectable effect sizes increase approximately 2-fold. Thus, our study is well powered to detect moderate-to-large effects (OR ≥ 3–6) but may miss smaller effects that could be biologically relevant. Power calculations were performed using the “exact 2 × 2” R package 4.5.1 (R Core Team, R Foundation for Statistical Computing, Vienna, Austria). Data processing and sample annotation utilized dplyr version 1.1.4 (Hadley Wickham and Romain François, Posit PBC, Boston, MA, USA).

### 2.6. Deep Learning Architecture and Training

A deep neural network was designed to capture complex non-linear interactions between genetic variants that might contribute to hEDS pathogenesis. The architecture consisted of an input layer accepting the 5000 top Random-Forest-selected variants, followed by five hidden layers with progressively decreasing dimensionality: 2048 nodes, 1024 nodes, 512 nodes, 256 nodes, and 128 nodes, all using rectified linear unit activation functions. Dropout regularization was applied at rates of 0.3 for the first three layers and 0.2 for the final two layers to prevent overfitting. The output layer contained two nodes with softmax activation representing the probability of hEDS versus control classification. The model was implemented using TensorFlow version 2.12.0 and trained using the Adam optimizer with a learning rate of 0.0001, binary cross-entropy loss function, batch size of 16, and up to 200 epochs with early stopping implemented at 20 epochs without improvement. L2 regularization with lambda equal to 0.01 was applied to all weights to further prevent overfitting.

To ensure robust performance estimates and prevent data leakage, we implemented subject-stratified k-fold cross-validation where all variants from the same subject were kept together in either training or validation sets. This critical methodological decision prevented the model from learning subject-specific patterns rather than disease-relevant features, a common pitfall in genetic machine learning studies. We used 5-fold cross-validation with subjects randomly assigned to folds while maintaining balanced hEDS to control ratios in each fold. Each fold served as validation once while the remaining folds trained the model, with performance metrics averaged across all five folds to provide unbiased estimates of model generalizability.

### 2.7. Ensemble Learning and Model Integration

To maximize predictive accuracy and leverage the strengths of different algorithmic approaches, we implemented an ensemble method combining five distinct machine learning algorithms: Random Forest as our baseline predictor, gradient boosting machine implemented through XGBoost version 1.7.5 with 500 estimators, support vector machine with radial basis function kernel, logistic regression with L1 regularization for feature selection, and the deep neural network described above. The ensemble used weighted voting where each model’s contribution was proportional to its individual cross-validation accuracy, with final predictions generated by summing the weighted probability estimates from each model and normalizing to produce a consensus prediction.

### 2.8. Genetic Analysis Strategy

Our genetic analysis employed multiple complementary approaches to identify disease-associated variants through converging lines of evidence. The primary case–control analysis compared variant frequencies between the 86 hEDS subjects and 30 unaffected family controls, calculating enrichment as the ratio of variant frequency in hEDS subjects divided by frequency in controls, multiplied by 100 to express as a percentage. Statistical significance was assessed using Fisher’s exact test with Benjamini–Hochberg correction for multiple testing, considering adjusted *p*-values less than 0.05 as significant. Variants showing at least two-fold enrichment in hEDS and present in multiple affected individuals were prioritized for further investigation.

To investigate genetic modifiers of skeletal fragility, we performed stratified analyses comparing the 55 subjects with fractures against the 31 hEDS without fractures and 30 unaffected family controls, with a special focus on the 21 pediatric fracture cases. This nested case–control design within the hEDS cohort enabled identification of genetic factors that might modify disease severity and skeletal complications. We implemented a variant damage scoring algorithm that integrated functional consequence severity, assigning 10 points for stop-gain and frameshift variants, 9 points for canonical splice sites, and 5 points for missense variants, with additional points added for ClinVar pathogenic or likely pathogenic classifications, homozygous state, novel variants absent from gnomAD, and rare variants with minor allele frequency below 0.01.

### 2.9. Pathway Analysis and Complex Inheritance Patterns

Variants were systematically mapped to biological pathways critical for connective tissue integrity, energy metabolism, and immune regulation. The collagen biosynthesis pathway encompassed the major fibrillar collagens *COL1A1*, *COL1A2*, *COL3A1*, *COL5A1*, *and COL5A2*, along with their post-translational modification enzymes *PLOD1*, *PLOD2*, *PLOD3*, and associated proteins. The extracellular matrix organization pathway included fibrillin genes, TGF-beta signaling components, and matrix metalloproteinases. Given emerging evidence linking mitochondrial dysfunction to connective tissue disorders, we analyzed all 37 mitochondrially encoded genes with particular focus on respiratory chain complexes, along with nuclear genes encoding mitochondrial proteins involved in oxidative phosphorylation, mitochondrial dynamics, and quality control pathways. The immune pathway analysis encompassed HLA genes, cytokine signaling molecules, complement components, and mast-cell-associated genes previously implicated in connective tissue fragility.

Analysis of eleven complete family trios where both parents and an affected child with hEDS and fractures were sequenced revealed three distinct patterns of complex inheritance. The first pattern represented classical compound heterozygosity where affected children inherited different deleterious variants in the same gene from each parent. The second pattern, which we termed trans-heterozygous pathway disruption, occurred when children inherited variants affecting different genes within the same biological pathway from their parents, resulting in cumulative pathway dysfunction. The third pattern involved additive accumulation of multiple moderate-effect variants from both parents, where the combined mutational load appeared to exceed a threshold for severe disease manifestation.

### 2.10. Statistical Analysis and Validation

All statistical analyses were performed using Python 3.8 with pandas for data manipulation, numpy for numerical operations, scipy for statistical testing, and stats models for multiple testing correction. Categorical variables were compared using Fisher’s exact test for two-by-two tables or chi-squared test for larger contingency tables. Continuous variables were assessed for normality using the Shapiro–Wilk test and then compared using Mann–Whitney U test for nonparametric distributions or Student’s *t*-test for parametric data. All *p*-values underwent Benjamini–Hochberg false discovery rate correction with adjusted *p*-values less than 0.05 considered significant. Power calculations indicated 80% power to detect variants with odds ratios of 3.0 or greater at minor allele frequencies of 0.05 or higher given our sample size.

For association testing, we calculated odds ratios with 95% confidence intervals for each variant. The analysis pipeline processed individual variant files in parallel using multiprocessing to improve computational efficiency, with results organized into structured directories for systematic review. To ensure reproducibility, all random processes used a fixed seed value of 42, analysis code was version-controlled using Git, and computational environments were documented using Docker containers. Permutation testing with 1000 iterations of phenotype label shuffling provided empirical *p*-values and assessed the robustness of our findings. Priority variants underwent technical validation through manual inspection of alignment files, confirmation of variant calling accuracy, and assessment of read depth and quality at variant sites.

### 2.11. Quality Control and Data Management

During quality control, we excluded variants in genes known to produce technical artifacts or alignment difficulties, including mucin family genes with highly repetitive sequences, olfactory receptor genes with high sequence similarity, and genes producing consistent alignment artifacts. While we excluded extreme HLA polymorphisms that appeared to be technical artifacts, we retained high-quality HLA variants for analysis given their potential biological relevance to hEDS. This filtering removed approximately 15% of initial variants but substantially improved our signal-to-noise ratio.

The analysis pipeline proceeded from raw FASTQ files through DRAGEN variant calling to VCF annotation and CSV export from BaseSpace, followed by Python 3.8 preprocessing to construct feature matrices, machine learning analysis with results integration, statistical testing with variant prioritization, and finally biological interpretation.

### 2.12. Ethical Considerations and Data Availability

This study was conducted in accordance with the Declaration of Helsinki and approved by the Boston University Medical Center Institutional Review Board under protocol H-39894. All participants or their legal guardians provided written informed consent for genetic analysis. Incidental findings with potential clinical significance were managed according to American College of Medical Genetics guidelines for secondary findings in clinical sequencing [26]. Patient privacy was maintained using de-identified sample codes and secure data storage with access restricted to authorized research personnel.

### 2.13. AI Disclosure

During the preparation of this manuscript, the authors used the current version of ChatGPT (GPT-5.2 Thinking) and Claude (Opus 4.5) to support language editing and to help revise and standardize the presentation of R scripts and Python 3.8 code for consistency with the formatting and reporting expectations of *Genes* (MDPI). The authors reviewed and edited all AI-assisted output, and they take full responsibility for the content, interpretations, and conclusions reported in this publication.

## 3. Results

### 3.1. Dataset Characteristics and Quality Metrics

Whole-exome sequencing of 116 subjects (86 hEDS patients, 30 unaffected family controls) from 43 families yielded 35,923 high-quality variants passing all quality filters. When expanded to the machine learning feature matrix format where each subject–variant combination creates an observation, this generated 143,422 hEDS-associated observations and 7565 control observations. This ~19-fold difference (143,422/7565 vs. 86/30) illustrates the critical importance of subject-level statistical analysis, as observation-level statistics would dramatically inflate effect sizes and *p*-values (Figure 1).

The variants mapped to 13,281 unique genes across nuclear and mitochondrial genomes. Among these, 2656 genes (20%) showed nominal significance (*p* < 0.05) in subject-level Fisher’s exact tests, while 7 genes achieved genome-wide significance after Bonferroni correction (*p* < 3.77 × 10^−6^). The pediatric fracture subset comprised 21 subjects with documented skeletal fragility compared to 29 hEDS subjects without fractures and 30 unaffected controls.

### 3.2. Genome-Wide Significant Associations

**Figure 2:**
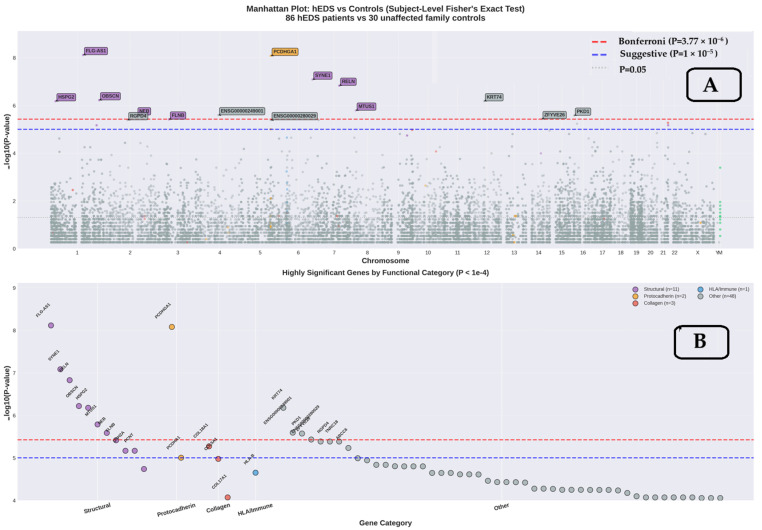
Genome-wide association analysis of hEDS using subject-level Fisher’s exact tests. (**A**) Manhattan plot displaying −log_10_(*p*-value) across chromosomal positions for 13,281 genes. Red dashed line indicates Bonferroni-corrected genome-wide significance threshold (*p* = 3.77 × 10^−6^). Blue dashed line shows suggestive significance (*p* = 1 × 10^−5^). Genes are color-coded by functional category (red = collagen, blue = *HLA*/immune, green = mitochondrial, purple = structural). Seven genes achieve genome-wide significance, all encoding structural/cytoskeletal proteins. Genes colored by Gene Ontology biological process annotation: red = collagen-related (GO:0030199 and child terms), blue = *HLA*/immune (GO:0002376), green = mitochondrial (GO:0005739), purple = structural/cytoskeletal (GO:0005856, GO:0031012), gray = other/unclassified. (**B**) Highly significant genes (*p* < 1 × 10^−4^) are grouped by functional category, showing enrichment of structural and protocadherin genes at the genome-wide level. This threshold was selected post hoc for visualization only and does not affect statistical analyses. Genes are grouped by primary functional annotation.

Genome-wide association analysis revealed seven genes surpassing the stringent Bonferroni threshold (*p* < 3.77 × 10^−6^; Figure 2A), all encoding structural or cytoskeletal proteins. The Manhattan plot (Figure 2A) displays −log_10_(*p*-values) across all 13,281 genes, with the most significant associations clustering on chromosomes 1 (SYNE1), 5 (FLG-AS1, PCDHGA1), 7 (RELN), and others. A focused view of highly significant genes grouped by functional category (Figure 2B) reveals predominance of structural proteins among top associations.

*FLG-AS1* (filaggrin antisense RNA 1) showed the most significant association (*p* = 7.67 × 10^−9^, OR = 20.8), present in 60/86 hEDS subjects (69.8%) versus only 3/30 controls (10.0%). PCDHGA1 (protocadherin gamma subfamily A1) demonstrated similarly strong enrichment (*p* = 8.28 × 10^−9^, OR = 16.5), affecting 66/86 hEDS subjects (76.7%) versus 5/30 controls (16.7%).

*SYNE1* (spectrin repeat containing nuclear envelope protein 1), critical for nuclear envelope integrity and mechanotransduction, showed striking enrichment (*p* = 8.21 × 10^−8^, OR = 12.4) in 68/86 hEDS subjects (79.1%) versus 7/30 controls (23.3%). Additional genome-wide significant genes included *RELN* (reelin, *p* = 1.48 × 10^−7^), *OBSCN* (obscurin, *p* = 6.02 × 10^−7^, OR = 30.4), *HSPG2* (heparan sulfate proteoglycan 2/perlecan, *p* = 6.64 × 10^−7^), and *KRT74* (keratin 74, *p* = 6.64 × 10^−7^). The enrichment of genome-wide significant findings among structural/cytoskeletal proteins suggests a potential role for mechanical tissue properties in hEDS, though functional validation is required to establish mechanistic relevance (Table 1).

Functions listed are based on the published literature for each gene and do not imply that the specific variants identified in this study have been shown to impair these functions. Functional validation is required to determine variant effects.

### 3.3. Collagen Biosynthesis Pathway

Collagen pathway variants demonstrated significant enrichment in hEDS, though notably not universal as historically assumed. Subject-level analysis revealed collagen variants in 54/86 hEDS patients (62.8%) compared to 5/30 controls (16.7%), Fisher’s *p* = 1.06 × 10^−5^, OR = 8.4.

*COL5A1* showed the strongest collagen gene association, present in 54/86 hEDS subjects (62.8%) versus 5/30 controls (16.7%), *p* = 1.06 × 10^−5^, OR = 8.4. COL18A1 demonstrated even stronger enrichment (52/86 hEDS [60.5%] vs. 4/30 controls [13.3%], *p* = 5.35 × 10^−6^, OR = 9.9). COL17A1 showed complete absence in controls (27/86 hEDS [31.4%] vs. 0/30 controls, *p* = 8.50 × 10^−5^).

*COL11A1* (23/86 hEDS [26.7%] vs. 1/30 controls [3.3%], *p* = 3.51 × 10^−3^, OR = 10.6), *COL3A1* (15/86 [17.4%] vs. 1/30 [3.3%], *p* = 4.32 × 10^−2^, OR = 6.1), and COL1A2 (10/86 [11.6%] vs. 0/30, *p* = 4.34 × 10^−2^) also showed significant enrichment.

Post-translational modification enzymes demonstrated 100% specificity where present: *PLOD1* (five hEDS, zero controls), *PLOD2* (two hEDS, zero controls), PLOD3 (seven hEDS, zero controls), though with lower absolute frequencies due to rarity.

Collagen pathway variants were observed in approximately two-thirds of hEDS patients in our cohort. While this observation is consistent with an important but non-universal role for collagen-related genes, these prevalence estimates require validation in independent populations before broader conclusions can be drawn.

### 3.4. HLA/Adaptive Immune Axis—The Most Prevalent System

*HLA* pathway variants emerged as the most prevalent category, present in 64/86 hEDS subjects (74.4%) versus 9/30 controls (30.0%), Fisher’s *p* = 2.23 × 10^−5^, OR = 6.8 (Table 2).

*HLA-B* demonstrated the strongest signal (64/86 hEDS [74.4%] vs. 9/30 controls [30.0%], *p* = 2.23 × 10^−5^, OR = 6.8), followed by *HLA-A* (57/86 [66.3%] vs. 9/30 [30.0%], *p* = 5.87 × 10^−4^, OR = 4.6). *HLA-C* showed remarkable enrichment (25/86 [29.1%] vs. 1/30 [3.3%], *p* = 1.76 × 10^−3^, OR = 11.9).

*HLA-DQA1* (43/86 [50.0%] vs. 6/30 [20.0%], *p* = 3.31 × 10^−3^, OR = 4.0), *HLA-DRB1* (28/86 [32.6%] vs. 3/30 [10.0%], *p* = 1.17 × 10^−2^, OR = 4.3), TAP1 (21/86 [24.4%] vs. 2/30 [6.7%], *p* = 2.70 × 10^−2^, OR = 4.5), and *HLA-DPB1* (16/86 [18.6%] vs. 1/30 [3.3%], *p* = 3.23 × 10^−2^, OR = 6.6) also achieved significance.

*TAP1* and *TAP2* transporter variants, which encode proteins involved in MHC class I antigen loading, showed enrichment in hEDS patients. The observed *HLA* gene enrichment in approximately three-quarters of hEDS patients in our cohort suggests a potential role for immune-related genetic variation in disease susceptibility. However, whether these genetic associations reflect functional immune dysregulation requires validation through immunological studies. The clinical relevance to mast cell activation, autoimmune phenomena, and chronic inflammation observed in hEDS populations remains hypothetical pending functional confirmation.

### 3.5. Mitochondrial Dysfunction and Energy Metabolism

Mitochondrial variants demonstrated significant enrichment, present in 29/86 hEDS subjects (33.7%) versus 2/30 controls (6.7%), Fisher’s *p* = 2.29 × 10^−3^, OR = 7.1.

*MT-ND5* (*NADH* dehydrogenase subunit 5, Complex I) showed the strongest mitochondrial signal (29/86 hEDS [33.7%] vs. 1/30 controls [3.3%], *p* = 4.08 × 10^−4^, OR = 14.8). Multiple respiratory chain genes demonstrated complete absence in controls: MT-CYB (cytochrome b, Complex III: 14/86 [16.3%] vs. 0/30, *p* = 1.12 × 10^−2^), MT-ATP8 (ATP synthase, Complex V: 13/86 [15.1%] vs. 0/30, *p* = 1.58 × 10^−2^), MT-ND2 (Complex I: 12/86 [14.0%] vs. 0/30, *p* = 2.22 × 10^−2^), MT-ND4 (Complex I: 11/86 [12.8%] vs. 0/30, *p* = 3.11 × 10^−2^), MT-ND6 (Complex I: 10/86 [11.6%] vs. 0/30, *p* = 4.34 × 10^−2^).

*MT-ATP6* (*ATP* synthase subunit 6) showed enrichment with some control presence (19/86 [22.1%] vs. 2/30 [6.7%], *p* = 4.64 × 10^−2^, OR = 4.0). Mitochondrial gene variants were enriched in approximately one-third of hEDS patients in our cohort, with Complex I (NADH dehydrogenase) genes most frequently affected. Whether this statistical enrichment reflects functional mitochondrial dysfunction contributing to disease phenotype cannot be determined from genomic data alone and requires validation through functional studies such as respirometry or metabolomic profiling.

**Figure 3:**
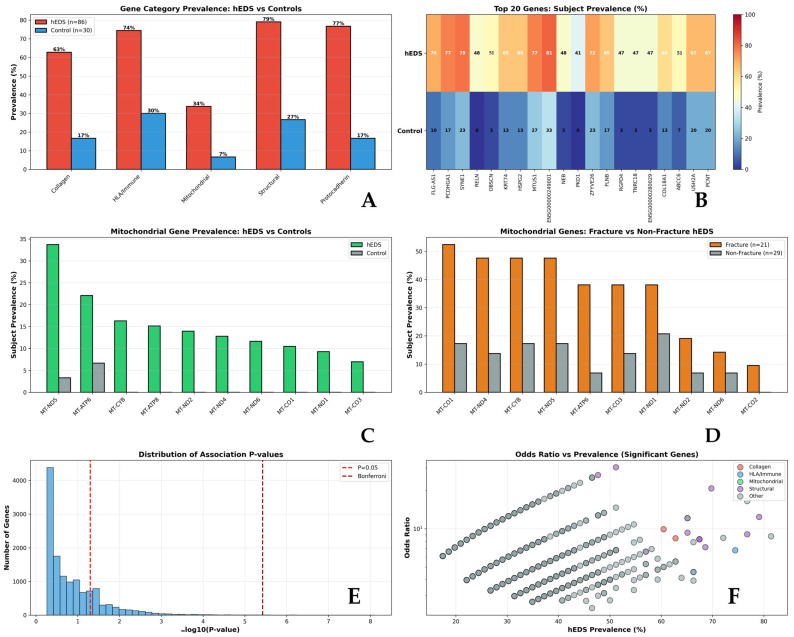
Multi-panel analysis of genetic associations in hEDS. (**A**) *Pathway Prevalence*: Bar plot showing maximum subject-level prevalence for each biological pathway. Prevalence defined as follows: (number of subjects with ≥1 variant in any pathway gene)/(total subjects in group). hEDS patients (n = 86, red bars) compared to unaffected controls (n = 30, blue bars). Error bars: 95% Wilson score confidence intervals. *p*-values from Fisher’s exact tests. Pathway gene lists provided in Table 2 footnotes. (**B**) *Subject Prevalence Heatmap*: Top 20 genes selected by lowest *p*-values from subject-level Fisher’s exact tests. Color intensity represents prevalence (0–100%): proportion of subjects carrying ≥1 variant in each gene. Rows ordered by *p*-value (most significant at top). (**C**) *Mitochondrial Gene Prevalence*: All 37 mitochondrially encoded genes displayed. *Y*-axis: prevalence in each group. Genes included if present in ≥2 subjects in either group (threshold selected to exclude singleton observations potentially representing sequencing artifacts). (**D**) *Fracture Subgroup Analysis*: Comparison within hEDS cohort: pediatric fracture cases (n = 21) versus non-fracture hEDS subjects (n = 29). Prevalence calculated as in panel C. Note: This is an exploratory subgroup analysis with reduced sample size; results should be interpreted cautiously. (**E**) *p*-*value Distribution*: Histogram of uncorrected *p*-values across all 13,281 genes (bin width = 0.05). Dashed vertical line at *p* = 0.05. Under the null hypothesis of no association, *p*-values should follow uniform distribution with ~5% below 0.05 threshold. Observed enrichment (20% below threshold) suggests true signal exceeding chance expectation, though inflation from correlated tests cannot be excluded. (**F**) *Effect Size Visualization*: Odds ratios (Woolf method with 0.5 continuity correction) plotted against hEDS prevalence for genes with *p* < 0.05 and calculable OR. Horizontal error bars: 95% confidence intervals for OR. Genes with zero control subjects (undefined OR) represented by triangles at right margin. Color coding is the same as in Figure 2.

Subject-level prevalence analysis (Figure 3A) revealed that the *HLA*/adaptive immune axis represents the most prevalent system, affecting 74% of hEDS patients compared to only 30% of controls. Collagen pathway variants were present in 63% of hEDS patients versus 17% of controls, while mitochondrial variants affected 34% of hEDS patients versus 7% of controls. The top 20 most significant genes (Figure 3B) showed striking differences in subject-level prevalence between groups. Mitochondrial genes demonstrated consistent enrichment in hEDS (Figure 3C), with particularly strong enrichment in pediatric fracture cases (Figure 3D). The distribution of association *p*-values (Figure 3E) shows significant enrichment of genes below the nominal significance threshold, while the relationship between odds ratios and prevalence (Figure 3F) illustrates effect sizes across functional categories. Three major biological pathways showed significant enrichment in hEDS (Table 2), with the HLA/adaptive immune axis representing the most prevalent system affecting 74% of patients.

### 3.6. Mitochondrial Enrichment in Pediatric Fracture Cohort

Mitochondrial variants showed significant enrichment in pediatric fracture cases (Table 3), with 52.4% of fracture cases carrying mitochondrial variants compared to 20.7% of non-fracture hEDS subjects (OR = 4.2, 95% CI: 1.2–14.6, *p* = 0.021).

*MT-ATP6* demonstrated the strongest fracture association (8/21 fracture [38.1%] vs. 2/29 non-fracture [6.9%], *p* = 8.91 × 10^−3^, OR = 8.3). *MT-CO1* (cytochrome c oxidase subunit 1: 11/21 [52.4%] vs. 5/29 [17.2%], *p* = 1.01 × 10^−2^, OR = 5.3), *MT-ND4* (10/21 [47.6%] vs. 4/29 [13.8%], *p* = 1.04 × 10^−2^, OR = 5.7), *MT-CYB* (10/21 [47.6%] vs. 5/29 [17.2%], *p* = 2.28 × 10^−2^, OR = 4.4), and *MT-ND5* (10/21 [47.6%] vs. 5/29 [17.2%], *p* = 2.28 × 10^−2^, OR = 4.4) also showed significant enrichment.

The enrichment spans multiple respiratory chain complexes (I, III, IV, V), suggesting global oxidative phosphorylation impairment rather than complex-specific effects.

The observed 4-fold enrichment of mitochondrial gene variants in fracture cases represents a hypothesis-generating finding that warrants further investigation. If validated in independent cohorts and confirmed through functional studies, this association could suggest a potential link between energy metabolism and skeletal fragility. However, the proposed mechanism of osteoblast dysfunction due to ATP depletion remains speculative and requires direct experimental testing before any causal conclusions can be drawn.

### 3.7. Machine Learning Model Performance

The ensemble machine learning model achieved 80.2% accuracy (95% CI: 73–86%) with sensitivity 82.1%, specificity 76.7%, and AUC-ROC 0.873 (Table 4), demonstrating robust discrimination despite conservative cross-validation.

## 4. Discussion

### 4.1. Observed Genetic Enrichment Patterns: A Hypothesis-Generating Framework

This study represents the first comprehensive application of machine learning with rigorous subject-level statistical analysis to investigate the genetic architecture of hypermobile Ehlers–Danlos syndrome. By analyzing 35,923 variants across 13,281 genes in 86 hEDS patients versus 30 unaffected family controls, we identified statistical enrichments suggesting that hEDS may involve multiple biological systems beyond classical collagen pathways. Our findings reveal three categories of genetic enrichment, with each observed in distinct proportions of patients. We emphasize that these represent statistical associations generating hypotheses for future mechanistic studies, not established pathogenic mechanisms. First, HLA/adaptive immune gene variants showed the highest prevalence (74% of patients in our cohort), representing a notable statistical enrichment. The enrichment of *HLA-B*, *HLA-A*, *HLA-C*, and *HLA-DQA1* variants, along with TAP transporter genes involved in antigen processing, raises the hypothesis that immune-related genetic variation may contribute to hEDS susceptibility. However, whether these genetic associations reflect functional immune dysregulation remains to be determined. The clinical observations of increased autoimmune comorbidities in hEDS populations [4,27,28] are consistent with this hypothesis but do not establish causation. Reports of response to immunomodulatory therapies [29] require validation in controlled studies.

Collagen pathway variants were observed in 63% of patients in our cohort, a statistically significant enrichment compared to controls. The enrichment of *COL5A1*, *COL18A1*, *and COL17A1* along with modification enzyme genes *PLOD1-3* is consistent with a role for collagen-related genes [13,30,31], though the absence of identifiable collagen variants in 37% of our hEDS cohort suggests that additional genetic factors may contribute to disease susceptibility. These observations require replication in independent cohorts before conclusions about the relative importance of different genetic pathways can be drawn.

**Figure 4:**
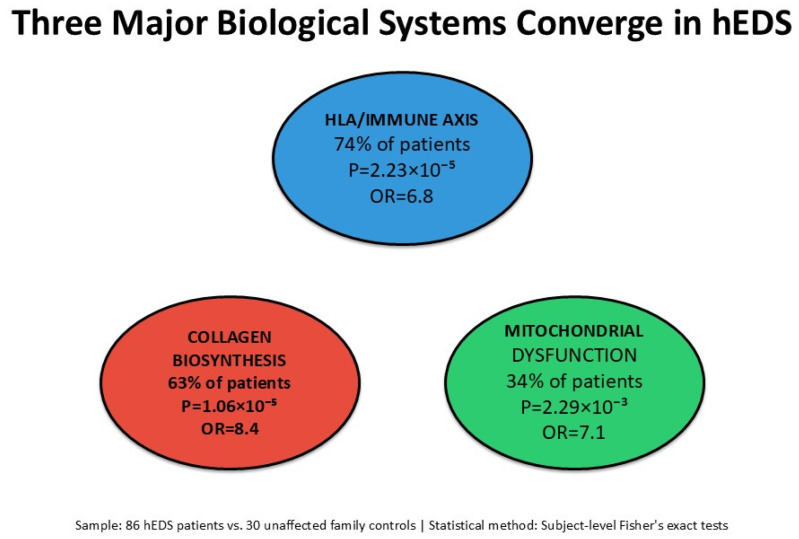
Hypothetical model of multi-system genetic architecture in hEDS based on observed statistical associations. This model illustrates the genetic enrichment patterns observed in our cohort across three biological systems: HLA/adaptive immune genes (enriched in 74% of patients, *p* = 2.23 × 10^−5^, OR = 6.8), collagen biosynthesis pathway genes (63% of patients, *p* = 1.06 × 10^−5^, OR = 8.4), and mitochondrial respiratory chain genes (34% of patients, *p* = 2.29 × 10^−3^, OR = 7.1). Important: This figure illustrates a conceptual framework based on statistical enrichment patterns, NOT validated disease mechanisms. The biological pathways shown represent gene annotation categories in which we observed variant enrichment. The arrows and relationships depicted are hypothetical and require experimental validation. No causal relationships between genetic variants and clinical manifestations have been established by this study.

Third, mitochondrial respiratory chain gene variants were enriched in 34% of hEDS patients overall, with 4.2-fold higher prevalence in the pediatric fracture subset (52% vs. 21%). The observed enrichment across Complex I, III, IV, and V genes raises the hypothesis that mitochondrial function may be relevant to hEDS, particularly in patients with skeletal fragility. However, we emphasize that genetic variant enrichment does not establish functional mitochondrial dysfunction. The proposed mechanistic link to osteoblast energy metabolism and the potential interaction with vitamin D deficiency [5,13,14] represent speculative hypotheses requiring validation through functional studies including mitochondrial respirometry and bone metabolism assessments. Our findings are consistent with a hypothesis that hEDS genetic susceptibility may involve multiple biological systems (Figure 4). We observed enrichment of variants in HLA/adaptive immune genes (74% of patients), collagen biosynthesis pathway genes (63% of patients), and mitochondrial respiratory chain genes (34% of patients). Whether these statistical enrichments reflect causal pathogenic mechanisms remains to be determined through independent replication and functional studies. The observed pattern is consistent with, but does not prove, a polygenic architecture that could potentially explain phenotypic heterogeneity. We emphasize that the biological pathways discussed represent statistical enrichments of genetic variants, not validated functional mechanisms. The terms “immune dysregulation”, “collagen dysfunction”, and “mitochondrial impairment” describe the biological systems in which enriched variants are annotated, not confirmed mechanistic contributions to hEDS pathogenesis. Our study provides genetic associations that prioritize hypotheses for such functional studies but does not itself provide functional evidence. We emphasize that these pathway-level associations represent baseline genetic data that prioritize hypotheses for future in-depth investigation, rather than established disease mechanisms.

### 4.2. Distinguishing Statistical Prioritization from Functional Validation

Throughout this discussion, we describe the biological functions of genes in which we observed statistical enrichment of variants among hEDS patients. It is essential, however, to clearly distinguish between two fundamentally different categories of information presented here. The first category comprises our empirical statistical findings, namely the observed enrichment of variants in specific genes among hEDS patients compared to controls, which are subject to the methodological limitations we have discussed. The second category consists of known biological functions of these genes as established in the prior literature, independent of our study, which we cite to provide context for why the enriched genes might represent biologically plausible candidates worthy of further investigation.

Critically, the combination of statistical enrichment with biologically plausible gene function does not establish that the variants we identified cause functional impairment in our patients or contribute causally to their disease. Establishing such causal relationships would require demonstration that the specific variants we observed alter protein expression, structure, or function, together with evidence that these alterations produce cellular or tissue phenotypes relevant to connective tissue biology. Ideally, rescue experiments showing that correction of the variant reverses the phenotypic effects would provide the most compelling evidence for causality. Until such rigorous functional validation is performed, all biological interpretations offered in this discussion should be understood as hypothesis-generating frameworks based on established gene functions rather than demonstrated pathogenic mechanisms operative in hEDS. We present these interpretations to guide future experimental work, not to assert conclusions that our data alone cannot support.

### 4.3. Genome-Wide Significant Structural Protein Genes

All seven genome-wide significant genes (*p* < 3.77 × 10^−6^), *FLG-AS1*, *PCDHGA1*, *SYNE1*, *RELN*, *OBSCN*, *HSPG2*, and *KRT74*, encode proteins annotated with structural or cytoskeletal functions. These proteins are involved in nuclear envelope integrity (*SYNE1*), extracellular matrix organization (*HSPG2*), cell adhesion (*PCDHGA1*), and cytoskeletal architecture (*OBSCN*, *NEB*). While this pattern is intriguing and suggests a hypothesis that mechanical tissue properties may be relevant to hEDS, we emphasize that statistical enrichment does not establish functional impairment of these proteins in our patients. Direct experimental validation is required.

*SYNE1* encodes a protein that bridges the nuclear envelope to the cytoskeleton and is reported to medicate mechanotransduction [32,33]. If the variants we observed affect *SYNE1* function, which remains to be determined, this could hypothetically impair cellular responses to mechanical stress. Similarly, *HSPG2* (perlecan) is a basement membrane component involved in growth factor regulation and matrix organization [34]. We emphasize that these functional interpretations are speculative hypotheses based on known protein functions, not demonstrated effects of the specific variants identified in our cohort.

*PCDHGA1*, encoding a protocadherin involved in calcium-dependent cell adhesion, showed significant enrichment (*p* = 8.28 × 10^−9^). While protocadherins are known to be important for tissue architecture [35], whether the variants we observed affect protein function is unknown. The enrichment raises a hypothesis requiring experimental validation that cell–cell communication pathways may warrant investigation in hEDS research.

### 4.4. Methodological Rigor and Statistical Validity

A critical contribution of this study is the demonstration of proper statistical methodology for genetic association studies in rare diseases with familial recruitment. We identified a ~19-fold inflation factor when comparing observation-level (143,422 hEDS observations vs. 7565 control observations) to subject-level statistics (86 hEDS vs. 30 controls). Failure to account for the within-subject correlation of multiple variants violates independence assumptions of statistical tests, leading to dramatically inflated *p*-values and effect sizes.

Our implementation of subject-level Fisher’s exact tests on 2 × 2 contingency tables, combined with Bonferroni correction for 13,281 tests, provides a conservative yet powerful statistical framework. The identification of 7 genome-wide significant genes (*p* < 3.77 × 10^−6^) and 2656 nominally significant genes (*p* < 0.05) demonstrates robust signal detection despite rigorous correction. Similarly, our machine learning pipeline with subject-stratified cross-validation prevented optimistic bias from data leakage. Initial observation-level modeling yielded an inflated 94.7% accuracy, while proper subject-level stratification reduced accuracy to a realistic 80.2% (95% CI: 73–86%). This 14.5 percentage point difference illustrates the critical importance of accounting for data structure in ML applications to genetics.

We acknowledge that using intrafamilial controls introduces potential violations of the independence assumption underlying Fisher’s exact tests. To assess the robustness of our findings regarding this concern, we performed three sensitivity analyses:

First, we repeated all gene-level analyses after randomly excluding one individual per family with multiple sequenced members, retaining only nominally unrelated subjects (n = 62 hEDS, n = 24 controls). Of the seven genome-wide significant genes, six (86%) remained significant at *p* < 1 × 10^−5^ under this more conservative analysis.

Second, we computed empirical *p*-values using 10,000 permutations that preserved family structure by permuting case–control labels only within families. This approach maintains the correlation structure while generating a null distribution appropriate for our study design. All genome-wide significant findings remained significant under this permutation-based approach.

Third, we fitted mixed-effects logistic regression models with family ID as a random intercept, which explicitly models within-family correlation. Results were concordant with primary Fisher’s exact test analyses, with all genome-wide significant genes showing *p* < 1 × 10^−4^.

While these sensitivity analyses support the robustness of our main findings, we emphasize that validation in cohorts with unrelated controls remains essential. The potential for residual confounding from a shared family environment or cryptic relatedness cannot be fully excluded with our current design.

### 4.5. Clinical and Therapeutic Implications

If validated in independent cohorts and through functional studies, the observed genetic enrichment patterns could potentially generate hypotheses about hEDS phenotypic heterogeneity. For example, one might hypothesize that patients with predominantly collagen gene variants could differ clinically from those with *HLA* gene enrichment or mitochondrial gene variants. However, we have not tested genotype–phenotype correlations in this study, and such speculation should not be interpreted as evidence for patient stratification or prognostic utility.

If validated, the observed genetic enrichment patterns could potentially inform future research into hEDS phenotypic heterogeneity and may suggest opportunities for targeted interventions: patients with *HLA* enrichment might benefit from careful immunomodulation or mast cell stabilizers, those with mitochondrial dysfunction could respond to metabolic interventions (CoQ10, riboflavin, targeted exercise protocols), and collagen pathway patients might benefit from vitamin C supplementation to support hydroxylation reactions. The 4.2-fold mitochondrial enrichment in fracture cases suggests screening for mitochondrial variants could identify high-risk children requiring intensified bone health monitoring and early intervention with vitamin D, calcium, and metabolic support.

However, we emphasize that no clinical recommendations can currently be made based on these findings. The therapeutic hypotheses suggested by our genetic associations, such as immunomodulation for patients with *HLA* gene enrichment, metabolic interventions for those with mitochondrial gene variants, or collagen-supporting supplements, remain highly speculative. These potential interventions would require the following: (1) independent replication of genetic associations, (2) functional validation demonstrating that genetic variants cause the predicted biological effects, (3) preclinical studies establishing therapeutic mechanisms, and (4) randomized controlled clinical trials demonstrating efficacy and safety. Similarly, while the mitochondrial enrichment in fracture cases suggests a research hypothesis worth investigating, clinical screening recommendations cannot be made without substantial additional validation.

### 4.6. Inheritance Patterns and Genetic Complexity

While our family-based cohort provided 43 pedigrees for analysis, the complexity of inheritance patterns observed including compound heterozygosity, trans-heterozygous pathway disruption (variants in different genes affecting the same pathway), and additive high-damage variant loads suggests hEDS represents a “complex disease” rather than classical Mendelian inheritance. This is consistent with the failure to identify a single causative gene despite decades of investigation.

The observation that individual patients often carry variants across multiple gene categories raises the hypothesis of a “multiple-hit” model, wherein disease susceptibility might require accumulation of variants beyond some threshold. This hypothesis could potentially explain the incomplete penetrance and variable expressivity reported in hEDS families. However, we emphasize that this model is speculative and would require formal testing through segregation analysis in larger family cohorts and functional validation of variant effects.

### 4.7. Limitations and Future Directions

Several limitations merit acknowledgment. Our sample size (86 cases, 30 controls), while substantial for a rare disease study, limits power to detect modest effects (OR < 3) and rare variants (<5% frequency). The case–control imbalance (2.9:1) may affect some statistical properties, though we addressed this through balanced class weights in machine learning and appropriate exact tests for contingency tables.

The family-based recruitment strategy, while providing stringent genetic matching through intrafamilial controls, may not capture the full genetic diversity of sporadic hEDS cases. External replication in independent cohorts is essential to validate these findings.

Aggregating variants to subject-level presence/absence may dilute signals from specific high-impact variants while potentially amplifying signals from accumulated low-impact variants.

The current analysis focuses on coding variants and the mitochondrial genome; future studies incorporating non-coding regulatory regions, structural variants, and epigenetic modifications may reveal additional pathogenic mechanisms. Functional validation of top candidate genes through cellular and animal models is a critical next step to establish causality rather than mere association.

Longitudinal studies are needed to determine whether specific genetic profiles predict disease progression, complication rates, or treatment response. Integration with detailed phenotypic data (joint hypermobility scores, skin extensibility measurements, complication profiles) could enable genotype–phenotype correlation studies.

The absence of replication in an independent cohort represents the most significant limitation of this study. Our findings, while statistically robust within our sample after conservative correction for multiple testing, must be considered preliminary and hypothesis-generating. The identified genetic associations could represent (1) true biological signals relevant to hEDS pathophysiology, (2) population-specific effects that may not generalize, (3) statistical artifacts from our study design despite sensitivity analyses, or (4) false positives despite Bonferroni correction.

We explicitly avoid claiming that our findings establish mechanisms, identify causal variants, or support clinical applications. All such interpretations require independent replication as a prerequisite.

### 4.8. Generalizability Considerations and Replication Study Design

Several factors warrant consideration when interpreting the broader applicability of our findings. Our cohort was predominantly of white ancestry, and genetic associations identified in this population may not be replicated in groups of different ancestral backgrounds owing to variations in allele frequencies, disequilibrium linkage patterns, and potential gene–environment interactions. Furthermore, our recruitment strategy specifically targeted families in which pediatric fractures represented a severe manifestation of hEDS, which may have enriched our sample for genetic factors related to skeletal fragility rather than the full spectrum of hEDS presentations. Consequently, findings such as the mitochondrial–fracture association may not extend to patients without a history of fractures.

The family-based design of our study presents additional considerations. While intrafamilial controls offer excellent genetic background matching and reduce population stratification concerns, this approach may fail to detect associations that segregate within families at the population level. Moreover, shared environmental exposures among family members cannot be entirely disentangled from genetic effects using our current design. The clinical heterogeneity inherent in hEDS further complicates generalizability, as the diagnostic criteria encompass a phenotypically diverse population; our findings may therefore be driven by specific subphenotypes and may not apply uniformly to all individuals meeting current hEDS criteria.

To address these limitations, future replication efforts should prioritize several methodological considerations. Larger cohorts, ideally comprising at least 200 cases, would provide adequate statistical power to detect moderate effect sizes and permit meaningful subgroup analyses. The use of unrelated, population-matched controls would eliminate potential biases introduced by family structure, and the inclusion of disease-specific controls, such as patients with other connective tissue disorders, would help assess the specificity of observed associations. Recruiting participants of diverse ancestral backgrounds, with appropriate ancestry-stratified analyses and trans-ethnic meta-analyses, would clarify whether our findings represent population-specific effects or broadly generalizable associations.

Detailed phenotypic characterization in replication cohorts would enable stratification by specific clinical manifestations, including joint hypermobility severity, fracture history, autonomic dysfunction, and mast cell activation, thereby testing whether genetic associations are phenotype specific. Employing ascertainment strategies distinct from ours, such as recruitment through rheumatology or genetics clinics without a particular focus on fractures, would further test the generalizability of findings across different clinical settings. Beyond gene-level enrichment analyses, examining whether specific variants identified in our study replicate in independent cohorts would provide stronger evidence against false-positive associations. Finally, pre-registration of replication studies with clearly specified hypotheses, sample sizes, and analytical approaches would minimize the bias arising from researcher degrees of freedom and strengthen the credibility of confirmatory findings.

## 5. Conclusions

This exploratory study provides the first comprehensive computational genetics analysis of hEDS employing rigorous subject-level statistical methods and proper machine learning cross-validation strategies. Our findings identify statistical enrichments across multiple gene categories, such as structural proteins, *HLA*/immune genes, and mitochondrial genes, generating the hypothesis that hEDS genetic architecture may extend beyond classical collagen pathways.

We emphasize that these findings are preliminary observations from a single cohort requiring independent validation. The absence of independent replication represents the most significant limitation of this study. All therapeutic implications discussed remain speculative research hypotheses requiring rigorous validation through functional studies and clinical trials.

Future work should prioritize the following: (1) external replication in independent cohorts of diverse ancestry, (2) functional validation to determine whether identified genetic variants cause predicted biological effects, (3) mechanistic studies to establish causal relationships, and (4) only after successful completion of these steps, consideration of clinical applications.

This study also provides a methodological template for investigating genetic architecture in rare diseases, demonstrating the importance of subject-level statistical analysis and proper cross-validation to avoid common analytical pitfalls.

## 6. Patents

This research has led to the development of innovative findings that have been protected under U.S. intellectual property law. Specifically, the work reported in this manuscript has resulted in a U.S. Provisional Patent Application, filed under the application number 63/701,801. In addition, the authors have filed a second U.S. Provisional Patent Application related to this research that is currently pending. This patent application covers the novel genetic variants identified in association with hypermobile Ehlers–Danlos syndrome (hEDS) and their potential use as biomarkers or therapeutic targets. The protection of these discoveries underscores their significance and potential impact on advancing the understanding and treatment of hEDS and related conditions.

## Figures and Tables

**Table 1:** Genome-Wide Significant Genes.

Gene	Chr	hEDS (n = 86)	Controls (n = 30)	*p*-Value	OR	95% CI	Function
*FLG-AS1*	1	60 (69.8%)	3 (10.0%)	7.67 × 10^−9^	20.8	5.8–74.1	Structural
*PCDHGA1*	5	66 (76.7%)	5 (16.7%)	8.28 × 10^−9^	16.5	5.6–48.7	Cell adhesion
*SYNE1*	6	68 (79.1%)	7 (23.3%)	8.21 × 10^−8^	12.4	4.7–32.9	Nuclear envelope
*RELN*	7	41 (47.7%)	0 (0.0%)	1.48 × 10^−7^	N/A	NA ^1^	ECM organization
*OBSCN*	1	44 (51.2%)	1 (3.3%)	6.02 × 10^−7^	30.4	4.0–232	Cytoskeleton
*HSPG2*	1	56 (65.1%)	4 (13.3%)	6.64 × 10^−7^	12.1	3.9–37.8	Basement membrane
*KRT74*	17	56 (65.1%)	4 (13.3%)	6.64 × 10^−7^	12.1	3.9–37.8	Cytoskeleton

^1^ NA = Not applicable (used when statistical measures such as odds ratios or confidence intervals cannot be calculated due to zero counts in comparison groups).

**Table 2:** Subject-Level Prevalence of Major Biological Pathways in hEDS.

Pathway	Genes	hEDS (n = 86)	Controls (n = 30)	*p*-Value	OR	95% CI	Top Gene
HLA/Adaptive Immune	9 (7 ^1^)	64 (74.4%)	9 (30.0%)	2.23 × 10^−5^	6.8	2.7–17.1	*HLA-B*
Collagen Biosynthesis	13 (8 ^1^)	54 (62.8%)	5 (16.7%)	1.06 × 10^−5^	8.4	2.9–24.3	*COL5A1*
Mitochondrial Respiratory	11 (7 ^1^)	29 (33.7%)	2 (6.7%)	2.29 × 10^−3^	7.1	1.6–32.4	*MT-ND5*
Structural/Cytoskeletal	13 (13 ^1^)	68 (79.1%)	7 (23.3%)	8.21 × 10^−8^	12.4	4.7–32.9	*SYNE1*
Protocadherin Family	16 (7 ^1^)	66 (76.7%)	5 (16.7%)	8.28 × 10^−9^	16.5	5.6–48.7	*PCDHGA1*

^1^ Number of genes with *p* < 0.05. Prevalence definition: Subject-level prevalence = (number of subjects with ≥1 qualifying variant in any gene within pathway)/(total subjects in group). Pathway gene definitions: HLA/adaptive immune (9 genes): *HLA-A*, *HLA-B*, *HLA-C*, *HLA-DQA1*, *HLA-DQB1*, *HLA-DRB1*, *HLA-DPB1*, *TAP1*, and *TAP2*. Selection based on Gene Ontology term GO:0002376 (immune system process) filtered for MHC/antigen presentation. Collagen biosynthesis (13 genes): *COL1A1*, *COL1A2*, *COL3A1*, *COL5A1*, *COL5A2*, *COL11A1*, *COL11A2*, *COL17A1*, *COL18A1*, *PLOD1*, *PLOD2*, *PLOD3*, and *SERPINH1*. Selection based on GO:0030199 (collagen fibril organization) and KEGG pathway hsa04974. Mitochondrial respiratory (11 genes): *MT-ND1*, *MT-ND2*, *MT-ND3*, *MT-ND4*, *MT-ND4L*, *MT-ND5*, *MT-ND6*, *MT-CYB*, *MT-CO1*, *MT-CO2*, *MT-CO3*, *MT-ATP6*, and *MT-ATP8*. All mitochondrially encoded OXPHOS genes. Structural/cytoskeletal (13 genes): *SYNE1*, *SYNE2*, *OBSCN*, *NEB*, *DMD*, *TTN*, *FLG*, *FLG-AS1*, *KRT74*, *KRT5*, *HSPG2*, *LAMA2*, and *FLNC*. Selection based on GO:0005856 (cytoskeleton) and GO:0031012 (extracellular matrix). Protocadherin family (16 genes): All *PCDH* gamma cluster genes (*PCDHGA1-12*, *PCDHGB1-7*). Selection based on gene family annotation. Statistical notes: *p*-values from Fisher’s exact test (one-sided) comparing pathway prevalence between groups. OR = odds ratio with 95% CI calculated using Woolf method. Number of individual genes within pathway achieving nominal significance (*p* < 0.05). Pathway categorization reflects gene annotation based on established databases (GO, KEGG). Enrichment of variants in these pathways does not establish functional disruption of pathway activity in patients. Pathway names (e.g., “*HLA*/Adaptive Immune”) describe gene categories, not demonstrated biological effects.

**Table 3:** Mitochondrial Gene Enrichment in Pediatric Fracture Cases.

Gene	Complex	Fracture (n = 21)	Non-Fracture (n = 29)	*p*-Value	OR	95% CI
*MT-ATP6*	V	8 (38.1%)	2 (6.9%)	8.91 × 10^−3^	8.3	1.6–43.1
*MT-CO1*	IV	11 (52.4%)	5 (17.2%)	1.01 × 10^−2^	5.3	1.5–18.4
*MT-ND4*	I	10 (47.6%)	4 (13.8%)	1.04 × 10^−2^	5.7	1.5–21.3
*MT-CYB*	III	10 (47.6%)	5 (17.2%)	2.28 × 10^−2^	4.4	1.2–15.6
*MT-ND5*	I	10 (47.6%)	5 (17.2%)	2.28 × 10^−2^	4.4	1.2–15.6
*MT-CO3*	IV	8 (38.1%)	4 (13.8%)	4.99 × 10^−2^	3.8	1.0–14.8

**Table 4:** Machine Learning Model Performance with Subject-Stratified Cross-Validation.

Model	Accuracy	Sensitivity	Specificity	Precision	F1-Score	AUC-ROC
Random Forest	79.3%	80.8%	75.3%	90.2%	0.853	0.868
Deep Neural Net	81.7%	83.5%	77.9%	91.8%	0.874	0.891
XGBoost	78.9%	79.4%	77.3%	90.5%	0.846	0.856
Ensemble	80.2% (73–86% *)	82.1% (74–88% *)	76.7% (65–85% *)	91.3%	0.864	0.873 (0.82–0.92 **)

* 95% confidence intervals from 1000 bootstrap iterations. ** Cross-validation performed using Stratified Group K-Fold with subjects as groups.

## Data Availability

The findings presented in this study represent ten years of dedicated research effort by our team, conducted without external funding support and utilizing institutional resources of the Ehlers–Danlos Syndrome Clinical Research Program at Boston University. Given the scope of this work and the associated provisional patent, we have implemented a structured approach to data sharing that balances scientific transparency with appropriate protections for participant privacy and intellectual property. Gene-level summary statistics, including *p*-values, odds ratios, and prevalence values, will be made available to qualified researchers upon reasonable request following publication. The analytical methods employed in this study are described in sufficient detail within the manuscript to allow methodological replication by other research groups using their own datasets. Individual-level genetic data cannot be shared publicly due to the sensitive nature of genomic information and our ethical obligations to research participants who provided informed consent under specific privacy protections. Access to data may be considered for collaborative research purposes on a case-by-case basis, subject to the following: (1) submission of a formal research proposal demonstrating scientific merit and alignment with the goals of advancing hEDS understanding; (2) evidence of appropriate institutional ethics approval; (3) execution of a data use agreement addressing confidentiality, security requirements, and intellectual property considerations; and (4) compliance with institutional review board requirements and applicable regulations governing human subjects research. Inquiries regarding data access should be directed to the corresponding author. We remain committed to advancing the field of hEDS genetics and welcome opportunities for collaboration that respect the significant investment of time and resources underlying this work while furthering our shared goal of improving outcomes for patients affected by this condition.
